# The Effect of Sacred Lotus (*Nelumbo nucifera*) and Its Mixtures on Phenolic Profiles, Antioxidant Activities, and Inhibitions of the Key Enzymes Relevant to Alzheimer’s Disease

**DOI:** 10.3390/molecules25163713

**Published:** 2020-08-14

**Authors:** Piya Temviriyanukul, Varittha Sritalahareuthai, Natnicha Promyos, Sirinapa Thangsiri, Kanchana Pruesapan, Wanwisa Srinuanchai, Onanong Nuchuchua, Dalad Siriwan, Nattira On-nom, Uthaiwan Suttisansanee

**Affiliations:** 1 Institute of Nutrition, Mahidol University, Salaya, Phuttamonthon, Nakhon Pathom 73170, Thailand; piya.tem@mahidol.ac.th (P.T.); varittha.sri@hotmail.com (V.S.); natnicha.prm@mahidol.ac.th (N.P.); poo.sweet@hotmail.com (S.T.); nattira.onn@mahidol.ac.th (N.O.-n.); 2 Food and Nutrition Academic and Research Cluster, Institute of Nutrition, Mahidol University, Salaya, Phuttamonthon, Nakhon Pathom 73170, Thailand; 3 Plant Varieties Protection Division, Department of Agriculture, Ministry of Agriculture and Cooperatives, Bangkok 10900, Thailand; kpruesapan@gmail.com; 4 National Nanotechnology Center (NANOTEC), National Science and Technology Development Agency(NSTDA), Klong Luang, Pathum Thani 12120, Thailand; wanwisa.sri@nanotec.or.th (W.S.); onanong@nanotec.or.th (O.N.); 5 Institute of Food Research and Product Development, Kasetsart University, Chatuchak, Bangkok 10900, Thailand; dalad.s@ku.th

**Keywords:** *Nelumbo nucifera*, sacred lotus, phenolics, antioxidant activities, Alzheimer’s disease, enzyme inhibitory properties

## Abstract

Sacred lotus (*Nelumbo nucifera*) has long been used as a food source and ingredient for traditional herbal remedies. Plant parts contain neuroprotective agents that interact with specific targets to inhibit Alzheimer’s disease (AD). Organic solvents including methanol, ethyl acetate, hexane, and *n*-butanol, are widely employed for extraction of sacred lotus but impact food safety. Seed embryo, flower stalk, stamen, old leaf, petal, and leaf stalk of sacred lotus were extracted using hot water (aqueous extraction). The extractions were analyzed for their bioactive constituents, antioxidant and anti-AD properties as key enzyme inhibitory activities toward acetylcholinesterase (AChE), butyrylcholinesterase (BChE), and β-secretase 1 (BACE-1). Results showed that the sacred lotus stamen exhibited significant amounts of phenolics, including phenolic acids and flavonoids, that contributed to high antioxidant activity via both single electron transfer (SET) and hydrogen atom transfer (HAT) mechanisms, with anti-AChE, anti-BChE, and anti-BACE-1 activities. To enhance utilization of other sacred lotus parts, a combination of stamen, old leaf and petal as the three sacred lotus plant components with the highest phenolic contents, antioxidant activities, and enzyme inhibitory properties was analyzed. Antagonist interaction was observed, possibly from flavonoids–flavonoids interaction. Further in-depth elucidation of this issue is required. Findings demonstrated that an aqueous extract of the stamen has potential for application as a functional food to mitigate the onset of Alzheimer’s disease.

## 1. Introduction

*Nelumbo nucifera* or sacred lotus in the Nymphaeceae family is a flowering perennial aquatic plant that grows extensively in Southeast Asia. Historically, the sacred lotus has been used for medicinal purposes as Ayurvedic and traditional Chinese medicine [1]. In Thailand, the sacred lotus has cultural importance as spiritual a symbol in Buddhism, and economic importance as ornamentation and horticulture to generate income. This aquatic plant is consumed as food and has wide-ranging uses as a folk medicine [2]. Various parts of the sacred lotus including leaves, flowers, stamens, embryos, and rhizomes were previously reported to promote health benefits [3,4,5,6,7]. These medicinal applications are the results of high health-promoting compounds, such as phenolic acids and flavonoids [3,4,5,6,7]. Sacred lotus leaves are rich in alkaloids, essential oils, organic acids, and flavonoids, especially quercetin [1,3,4,8]. Stamens are abundant in flavonols, including kaempferol, myricetin, quercetin, isorhamnetin, and their glycosides [1,3,4,8], while flavonoids and anthocyanidins are mostly found in the flowers. Moreover, alkaloids, procyanidins, polyphenols, and polysaccharides are highly presented in sacred lotus seeds. 

All sacred lotus parts contain high bioactive compounds, and have been used for pharmacological purposes such as anti-oxidative, astringent, emollient, diuretic [6], anti-diabetic [8], anti-hyperlipidemic [9], anti-aging [6,10], anti-ischaemia [11], anti-viral [12], anti-inflammatory [13,14], anti-allergic [15], anti-cancer [16], and hepatoprotective effects [17]. Interestingly, sacred lotus extracts were also previously reported to affect neurodegenerative disorders, especially Alzheimer’s disease (AD) [18,19,20]. This neurodegenerative disorder is a form of dementia presented in the elderly and is expected to increase because of the growing aging society. AD is characterized by loss of memory and personality, eventually resulting in reduced ability to perform normal daily activities, with death of patients within 3 to 9 years [21]. Several biological pathways have been proposed for AD pathogenesis. A low level of the cholinergic neurotransmitter, acetylcholine, correlates with cognitive impairments in AD cases; hence, inhibition of the acetylcholinesterase (AChE) and butyrylcholinesterase (BChE), the acetylcholine degrading enzymes, might delay AD onset. Besides, the amyloidogenic pathway is thought to contribute to AD, leading to the accumulation of amyloid peptides (Aβ peptides) and senile plaques. Brain biopsies of AD patients and rat models exhibited accumulation of amyloid plaques in the hippocampus and cerebral cortex as regions responsible for memory and cognitive functions [22]. The amyloid precursor protein (APP) is cleaved by beta-site amyloid precursor protein cleaving enzyme 1 or beta-secretase 1 (BACE-1), resulting in cytotoxic Aβ peptides-mediated oxidative stress and neuronal cell death. Increased oxidative stress also plays an important role in AD pathogenesis, and enhanced alteration of the antioxidant enzyme expression and activity of catalase and superoxide dismutase (SOD) in both the central and peripheral nervous system of AD cases [23]. Currently, many plant extracts have been reported to exhibit anti-AD properties through inhibition of these biological pathways of AD occurrence. Interestingly, it was previously suggested that methanolic extract of sacred lotus stamen expressed psychopharmacological effects by an increase in dopaminergic or norephinergic neurotransmission through cAMP formation activation [24]. Stamen extracts were also found to increase local cholinergic neurotransmitters through inhibition of AChE [18], while sacred lotus seeds exhibited inhibitory activity toward AChE, BChE, and BACE-1 [20]. Treatment with hydroethanolic extract of sacred lotus flowers to stress-induced rats also ameliorated brain damage, memory deficit, and oxidative stress but inhibited both monoamine oxidases and AChE in the rat hippocampus [25]. Previous data suggest that sacred lotus could be used as a potential neuroprotective agent, especially for AD treatment [26].

Although all parts of the sacred lotus plant have medicinal applications, particularly anti-AD properties, only rhizomes and seeds are widely consumed by Asians [6]. Utilization of other parts of the sacred lotus remains low, with limited food applications. Organic solvents including methanol, hexane, acetone, and toluene are mostly employed to perform extractions; however, these chemicals are toxic and unsuitable for food applications. Therefore, here, aqueous extracts were performed to investigate and compare antioxidant activities, bioactive constituents, and anti-AD properties of each part of the sacred lotus plant. Anti-AD properties were also investigated using a combination of other parts of the sacred lotus. This research promoted functional food development to combat AD in the future.

## 2. Results

### 2.1. Optimization of Extraction Conditions

Optimizing the extraction conditions of medicinal plants, especially the sacred lotus, is important to maximize the yield of bioactive compounds and promote health benefits [27,28]. Extraction of bioactive compounds with antioxidant activities is affected by many factors including extraction temperature and shaking time, along with interaction capability between substance particles and solvent [29]. The stamen of the sacred lotus was chosen as the representative of all plant parts. Extraction of sacred lotus stamen is generally conducted using organic solvents, including methanol, hexane, acetone, and toluene, which are unsuitable for food applications. Therefore, here, aqueous extracts of sacred lotus stamen were optimized and determined for total phenolic contents (TPCs) and antioxidant activities using the ferric ion reducing antioxidant power (FRAP) assay to assess extraction efficacy. Extraction parameters including material-to-solvent ratio (extract concentration), extraction temperature, and shaking time were also investigated.

Under fixed extraction temperature at 50 °C and shaking time of 2 h, sacred lotus stamen was extracted with concentrations ranging from 10 to 50 mg/mL. Results suggested that TPCs at extract concentrations of 10 and 20 mg/mL were significantly higher (52.98–56.52 mg gallic acid equivalent (GAE)/g dry weight (DW)) than extracts at concentrations of 30–50 mg/mL (37.40–44.40 mg GAE/g DW) (Table 1). Similar results were observed in antioxidant activities determined by FRAP assay. Extract concentration of 10 mg/mL exhibited significantly higher FRAP activity (731.80 µmol Trolox equivalent (TE)/g DW) than higher extract concentrations (420.70–656.81 µmol TE/g DW). Therefore, extract concentration of 10 mg/mL was chosen for further investigation of extraction temperature and shaking time parameters.

Under the fixed extract concentration at 10 mg/mL and extraction temperature of 50 °C, sacred lotus stamen was extracted utilizing different shaking times ranging from 0.5 to 4 h. The TPCs were slightly affected by different shaking times (54.64–62.09 mg GAE/g DW); however, FRAP activities were different under these conditions (Table 2). Reaction shaking for 1 h exhibited the significantly highest FRAP activity (722.98 µmol TE/g DW) than the other shaking time periods (655.02–661.56 µmol TE/g DW). Thus, shaking time at 1 h was chosen for further investigation of optimized extraction temperature.

Under fixed extract concentration of 10 mg/mL and shaking time for 1 h, sacred lotus stamen was extracted utilizing different extraction temperatures ranging from 30 to 90 °C. Results suggested that extraction at high temperatures (70–90 °C) exhibited significantly higher TPCs (60.17–64.27 mg GAE/g DW) than lower temperatures of 30–50 °C (TPCs of 51.19–51.98 mg GAE/g DW) (Table 3). Similar results were observed with FRAP activities. Extraction under a high temperature of 90 °C gave higher antioxidant activity (742.75 µmol TE/g DW) than extractions at lower temperatures (646.04–697.49 µmol TE/g DW). Thus, optimized extraction temperature was 90 °C.

Thus, optimized extraction conditions of sacred lotus stamen, performed under aqueous-based extraction (ultrapure water) were 10 mg/mL extract concentration, 90 °C extraction temperature, and 1-h shaking time to achieve the highest TPCs and FRAP activities. 

### 2.2. Total Phenolic Contents (TPCs), Total Anthocyanin Contents (TACs), and Phenolic Profiles

The TPCs of sacred lotus extracts ranged from 2.75 to 39.09 mg GAE/g DW (Table 4). Among different sacred lotus parts, old leaf exhibited the significantly highest TPC, followed by stamen, seed embryo, petal, flower stalk, and leaf stalk, respectively. However, total anthocyanin contents (TACs) were only detected in stamen (0.23 mg cyanidin 3-*O*-glucoside equivalent (C3GE)/g DW) and petal (0.05 mg C3GE/g DW).

The HPLC analysis suggested different types and quantities of phenolics in each sacred lotus part (Table 4 and Supplementary Figures S1–S5). For phenolic acids, gallic acid was found in old leaf, petal, and leaf stalk in the range of 49.38 to 277.84 mg/100 g DW, while none was detected in seed embryo, flower stalk, and stamen. The highest gallic acid content was detected in petal, with lowest in old leaf. Ferulic acid was only detected in seed embryo (24.71 mg/100 g DW), while p-coumaric acid was detected in seed embryo (105.34 mg/100 g DW) and stamen (10.78 mg/100 g DW). Interestingly, various flavonoids were detected in sacred lotus extracts. Naringenin was the most abundant flavanone with content ranging from 1064.17 to 2241.51 mg/100 g DW. Seed embryo, flower stalk, stamen, and petal exhibited significantly higher naringenin contents than leaf stalk and old leaf, respectively. Quercetin (35.95–458.56 mg/100 g DW) and isorhamnetin (2.67–237.85 mg/100 g DW) were also detected in all sacred lotus parts. Old leaf exhibited the highest quercetin contents, followed by petal, seed embryo, flower stalk, stamen, and leaf stalk, respectively. The highest isorhamnetin content was observed in petal, followed by stamen, seed embryo, leaf stalk, flower stalk, and old leaf, respectively. Kaempferol (3.87–197.83 mg/100 g DW) was detected in all sacred lotus parts, except leaf stalk. The highest content of kaempferol was detected in petal, followed by stamen, flower stalk, seed embryo, and old leaf, respectively. Luteolin (4.89–37.50 mg/100 g DW) was detected in seed embryo, flower stalk, and leaf stalk, while myricetin (7.63–8.89 mg/100 g DW) was detected in flower stalk, stamen, and petal. Interestingly, two anthocyanidins, including cyanidin (7.15–1901.52 mg/100 g DW) and delphinidin (6.15–1837.27 mg/100 g DW), were detected in all sacred lotus parts. The highest cyanidin content was detected in seed embryo, followed by petal, old leaf, stamen, flower stalk, and leaf stalk, respectively, while petal contained the highest delphinidin content, followed by seed embryo, stamen, old leaf, flower stalk, and leaf stalk, respectively.

### 2.3. Antioxidant Activities 

Under optimized extraction conditions, antioxidant activities of sacred lotus extracts were determined using 2,2-diphenyl-1-picrylhydrazyl (DPPH) radical scavenging activity, ferric ion reducing antioxidant power (FRAP), and oxygen radical absorbance capacity (ORAC) assays (Table 5). Results suggested that all sacred lotus extracts exhibited DPPH radical scavenging activities in the range of 0.43–1.21 µmol TE/100 g DW, FRAP activities in the range of 15.39–281.46 µmol TE/g DW, and ORAC activities in the range of 119.94–1001.12 µmol TE/g DW. Old leaf exhibited the significantly highest DPPH radical scavenging and ORAC activities, followed by stamen, petal, seed embryo, flower stalk, and leaf stalk, respectively. Antioxidant activities determined by FRAP assay suggested that stamen exhibited significantly highest antioxidant activity, followed by old leaf, seed embryo, petal, flower stalk, and leaf stalk, respectively. Thus, the top three sacred lotus parts with highest overall antioxidant activities were stamen, old leaf, and petal.

Since antioxidants can fight against oxidative stress in different mechanisms, including single electron transfer (SET) and hydrogen atom transfer (HAT), mixtures of the three sacred lotus parts (stamen, old leaf, and petal) with the highest overall antioxidant activities were investigated to establish the best combination with high antioxidant activities and TPCs that surpassed individual properties. Mixtures were prepared using different ratios (0 to 3) as indicated in Table 6. The TPCs of all mixtures ranged from 6.73–43.44 mg GAE/g DW. The mixture of stamen, old leaf, and petal extract at ratio 0:3:0 (or only old leaf) exhibited the highest TPC, while the mixture of stamen, old leaf, and petal extract at ratio 0:0:3 (or only petal) exhibited the lowest. Antioxidant activities determined by DPPH radical scavenging assay ranged 0.76–1.12 µmol TE/100 g DW. The mixture of stamen, old leaf, and petal extract at ratio 3:0:0 (or only stamen) exhibited the highest DPPH radical scavenging activity, while the mixture of stamen, old leaf, and petal extract at ratio 0:0:3 (or only petal) exhibited the lowest. Likewise, the FRAP activities ranged 54.58–296.50 µmol TE/g DW. The mixture of stamen, old leaf, and petal extract at ratio 3:0:0 (or only stamen) exhibited the highest FRAP activity, while the mixture of stamen, old leaf, and petal extract in the ratio of 2:2:3 exhibited the lowest. The ORAC activities ranged 212.26–670.83 µmol TE/g DW. The mixture of stamen, old leaf, and petal extract at ratio 1:3:3 exhibited the highest ORAC activity, while the mixture of stamen, old leaf, and petal extract at ratio 0:0:3 (or only petal) exhibited the lowest. 

### 2.4. In Vitro Inhibitory Activities of the Key Enzymes Relevant to Alzheimer’s Disease

Sacred lotus extracts inhibited the key enzymes relevant to AD, including acetylcholinesterase (AChE), butyrylcholinesterase (BChE), and beta-secretase (BACE-1) (Table 7). The AChE inhibitory activities of all sacred lotus extracts ranged from 55.75–90.70% inhibitions using extract concentration of 10 mg/mL. The half-maximal inhibitory concentration (IC_50_) suggested that stamen and old leaf were the strongest inhibitors against AChE with the lowest IC_50_ values of 0.66 and 1.09 mg/mL, respectively while leaf stalk was the weakest inhibitor with the highest IC_50_ value of 9.47 mg/mL (Supplementary Figure S6). Likewise, all sacred lotus extracts exhibited BChE inhibitory activities in the range of 34.72–93.72% inhibitions using extract concentration of 10 mg/mL. Stamen with the lowest IC_50_ value against BChE (0.08 mg/mL) was the strongest inhibitor, while seed embryo, flower stalk, and leaf stalk were the weakest inhibitors with the highest IC_50_ values ranging from 10.80 to 11.94 mg/mL (Supplementary Figure S7). Interestingly, BACE-1 inhibitory activities of all sacred lotus extracts ranged 44.51–75.61% inhibitions using extract concentration of 10 mg/mL. Flower stalk and leaf stalk exhibited the highest inhibitory activities, while seed embryo exhibited the lowest.

Since stamen, old leaf, and petal potentially exhibited the highest inhibitory activities against the key enzyme relevant to AD, mixtures of these sacred lotus parts (different ratios ranging from 0 to 3) were investigated to establish the best combination with the highest potential to combat AD key enzymes (Table 8). The AChE inhibitory activities of all mixtures ranged 13.66–80.58% inhibitions using extract concentration of 0.9 mg/mL. The mixture of stamen, old leaf, and petal extract at ratio 3:0:0 (or only stamen) and the mixture at ratio 0:3:0 (or only old leaf) exhibited the highest AChE inhibitory activities, while the mixture at ratio 1:3:2 exhibited the lowest. Likewise, the BChE inhibitory activities of all mixtures ranged 17.36–99.30% inhibitions using extract concentration of 0.9 mg/mL. The mixture of stamen, old leaf, and petal extract at ratio 3:0:0 (or only stamen) exhibited the highest BChE inhibitory activity, while the mixture at ratio 1:3:2 exhibited the lowest. Inhibitory activities against BACE-1 of all mixtures ranged 44.29–68.12% inhibitions using extract concentration of 9 mg/mL. However, various mixtures of sacred lotus parts, including recipe Nos. 10, 18, 19, 24, 26, 27, and 28 (the mixtures at ratios 1:3:1, 2:2:1, 2:3:1, 2:3:2, 3:2:1, 3:2:3, 3:3;1, and 3:3:2 (stamen: old leaf: petal), respectively), exhibited high BACE-1 inhibitory activities, while the mixture at ratio 0:0:3 (or only petal) exhibited the lowest.

## 3. Discussion

Antioxidant activities, bioactive constituents, and anti-AD properties of *Nelumbo nucifera* or sacred lotus targeting acetylcholinesterase (AChE), butyrylcholinesterase (BChE), and beta-secretase (BACE-1) were determined and compared. Each part of the sacred lotus plant including seed embryo, flower stalk, stamen, old leaf, petal, and leaf stalk was investigated. Previous data suggested that sacred lotus extracted by organic solvents may delay AD onset; however, using organic solvents such as methanol, hexane, or toluene for food applications is undesirable. Therefore, each part of the sacred lotus plant was extracted using hot water (aqueous extraction). Results indicated that the sacred lotus extracts contained phenolic acids, including gallic acid, ferulic acid, and *p*-coumaric acid, and flavonoids, including naringenin, quercetin, isorhamnetin, kaempferol, luteolin, myricetin, cyanidin, and delphinidin, in different amounts, depending on each sacred lotus plant part. Total phenolic contents (TPCs) and total anthocyanidin contents (TACs) also indicated that stamen, petal, and old leaf extracts contained the highest phenolic contents, with high antioxidant activities and inhibitory activities against the key enzyme relevant to AD. Lastly, combinations of different sacred lotus parts were also examined as a preliminary study of the combined effect of each part that might contribute to functional food development to combat AD in the future.

Results showed that the sacred lotus plant extracts contained several bioactive compounds. The TPCs were the highest in old leaf (39.09 ± 0.79 mg GAE/g DW), followed by stamen (36.37 ± 0.73 mg GAE/g DW) and petal (12.25 ± 0.36 mg GAE/g DW), while the lowest TPC was detected in leaf stalk (2.72 ± 0.10 mg GAE/g DW) (Table 4). These results concurred with an earlier report [30]. Another study showed that methanolic extract of *n. nucifera* cultivars “Sattabut” and “Sattabongkoj” grown in Northeastern Thailand exhibited the highest TPCs in the seedpod (77.00 ± 6.22 to 109.90 ± 4.37 mg GAE/g DW), stamen (84.90 ± 3.51 to 88.20 ± 6.49 mg GAE/g DW), and leaf (61.90 ± 2.29 to 73.20 ± 6.10 mg GAE/g DW). These results indicated that methanol possessed higher extraction efficacy for phenolic acids than hot water. Similar results were also reported by Lee and colleagues [31]. Nevertheless, other than the extraction solvent, differences in results might be due to the variations in extraction methods, lotus cultivars, parts of lotus, and growing environment. The HPLC analysis indicated different contributions of phenolic acids and flavonoids in each part of the sacred lotus plant. Considering phenolic acids, TPCs were high in aqueous fractions of old leaf and stamen, with only small amounts of gallic acid, ferulic acid, and *p*-coumaric acid detected. By contrast, gallic acid was the major phenolic acid in both sacred lotus petal and leaf stalk (Table 4). Flavonoids were more abundant than phenolic acids, particularly naringenin, that was high in all sacred lotus parts, while trace amounts of myricetin and luteolin were detected (Table 4). Interestingly, most of the detected flavonoids shown in Table 4 were hypothesized to be bioproducts of naringenin during flavonoid biosynthesis. The *O*-glycosylation of naringenin leads to apigenin and luteolin, while *C*-glycosylation of naringenin results in the creation of syringetin, diosmetin, kaempferol, isorhamnetin and quercetin [32]. Data indicated that *O*-glycosylation of naringenin was low compared to *C*-glycosylation as small amounts of luteolin and under-detected apigenin were reported in all sacred lotus parts. The naringenin presented in old leaf may be sequentially metabolized by flavanone 3-hydroxylase (F3H), flavonoid 3′ hydroxylase (F3/H) and flavonol synthase (FLS), leading to quercetin production. Similarly, naringenin catalyzed mainly by F3H and FLS produces kaempferol and isorhamnetin, which were prominent in sacred lotus stamen and petal. Anthocyanins were only detected in the red cultivar of lotus and not in the white cultivar [33]. Two anthocyanidins, cyanidin and delphinidin, were observed in all sacred lotus parts (Table 4). This could be the result of a sequential reaction of naringenin by F3H and anthocyanidin synthase (ANS) [33]. Consistent with the former data, the bioactive constituents in sacred lotus varied greatly according to the types of tissue, genetic background, and extraction methods [1]. Furthermore, flavonoid contents could be used as a potential database for *N. nucifera* authentication [34].

Antioxidant ability to scavenge free radicals of aqueous extracts of sacred lotus was evaluated by two major antioxidant mechanisms of single electron transfer (SET) measured by DPPH radical scavenging and FRAP assays, and hydrogen atom transfer (HAT) measured by ORAC assay [35]. The three sacred lotus parts with the highest antioxidant activities were stamen, old leaf, and petal (Table 5). This correlated well with their bioactive constituents. A similar trend of stamen and leaf possessing significant antioxidant activity in SET mechanism was also previously reported. Lotus leaf and stamen extracts recorded the IC_50_ values of DPPH radical scavenging assay at 20.7 and 42 µg/mL, respectively compared with L-ascorbic acid at 4.82 µg/mL [36]. On the other hand, the ORAC assay in HAT mechanism is based on interactions between the peroxyl radical and a fluorescein probe to produce non-fluorescent fluoresceinyl radicals, while antioxidants of interest act as the competitive inhibitors. Thus, if the plant extract contains high antioxidants, the decrease in fluorescent detection as reaction kinetics will be slower than the one with low or no antioxidants. Comparing to the strong antioxidant, L-ascorbic acid, with the ORAC value of 9350 µmol TE/g [37], different parts of sacred lotus extracts (Table 5) and their mixtures (Table 6) contained lower antioxidant activities in this mechanism. Nevertheless, flavonoids prefer HAT based reactions to quench free radicals rather than SET based reactions [38]. However, the data in Table 5 demonstrated that flavonoids or other bioactive compounds may also contribute to the SET based reactions. In agreement with our data, Lin and colleagues [5] showed that flavonoids mainly contributed to antioxidant activities in sacred lotus compared to other phenolics. Further investigation revealed that a mixture of stamen, old leaf, and petal extracts at ratio 3:0:0 (or only stamen) exhibited the highest DPPH radical scavenging and FRAP activities, while a mixture of stamen, old leaf, and petal extracts at ratio 1:3:3 exhibited the highest ORAC activity (Table 6). A detailed analysis of recipe Nos. 4, 5, 6 or 13, 14, 15 or 21, 22, and 23 suggested an increase in petal ratio (1 to 3), while stamen and leaf ratios remained fixed. Addition of petal extract exhibited antagonist effects measured by ORAC assay, which relied on the ability to quench cellular radical oxidants [39], suggesting that petal extract may contain antagonistic agents. Conversely, evidence of a synergistic effect of antioxidant activities among these three sacred lotus parts was lacking, since both synergistic and antagonistic interactions occurred between flavonoids [40].

Phytochemicals in sacred lotus including phenolics and aporphine alkaloids have been shown to inhibit the key enzymes relevant to AD, including AChE and BChE [41,42]. However, alkaloids are poorly water-soluble compounds, thereby the anti-AChE and BChE activities in this study may depend on phenolics. Phenolic contents also corresponded to the inhibition of the key enzymes relevant to AD, including AChE and BChE. The three sacred lotus parts with the highest AChE and BChE inhibitions were stamen, old leaf, and petal (Table 7). Inhibitory activities of these enzymes were related to the presence of phenolics in sacred lotus. The most abundant flavonoid, naringenin, was previously reported to exhibit the IC_50_ values of 42.66 µM against AChE and >100 µM against BChE [43]. The second most abundant flavonoid, quercetin, was determined as a stronger inhibitor with the IC_50_ value of 3.60 µM against AChE [44] and 1.2 times higher IC_50_ value against BChE than AChE inhibition [45]. Other minor flavonoids included isorhamnetin (the IC_50_ value of 24.18 µM against AChE [46], while no reports detailed its BChE inhibition), kaempferol (the IC_50_ value of 3.05 µM against AChE [44] and slightly lower IC_50_ value against BChE using the same concentration at 1 mM [41]), luteolin (the IC_50_ values of 88.04 µM against AChE [47] and 129.96 µM against BChE [47]), myricetin (similar IC_50_ values against AChE and BChE as kaempferol [41]), cyanidin (14.43 µM against AChE [44] and similar IC_50_ value against BChE using the same concentration at 1 mM [41]), and delphinidin (the IC_50_ value of 44.67 µM against AChE [44] and similar IC_50_ value against BChE using the same concentration at 1 mM [41]) were also previously reported with different degrees of AChE and BChE inhibitions. Thus, stamen with high phenolic contents was the strongest AChE and BChE inhibitor with the lowest IC_50_ values. Old leaf and petal as the second and third strongest inhibitors were chosen to mix with stamen to investigate if the combination had even higher AChE and BChE inhibitions. Results, however, suggested that individual extracts (stamen and old leaf) exhibited higher AChE and BChE inhibitory activities than combinations of the three sacred lotus parts (Table 8). Paired combinations of phenolic acids were previously suggested to exhibit lower AChE and BChE inhibitory activities than the sum of the individual inhibitory activities [41]. Similar results were observed with pairs of phenolic acids and flavonoids [41], suggesting that the lack of synergistic effect within and between phenolic acid and flavonoids, as well as the accommodation of two phenolics might not fit the small capacity catalysis pocket of both enzymes. Moreover, a previous study reported that aporphine alkaloids isolated from *N. nucifera* exhibited anti-AChE activities.

Inhibitory activities against another key enzyme in AD occurrence, BACE-1, suggested different results from AChE and BChE inhibitions, with flower stalk and leaf stalk exhibiting the highest BACE-1 inhibitory activities (Table 7). These outcomes led to various mixtures of sacred lotus parts including recipe Nos. 10, 18, 19, 24, 26, 27, and 28 that exhibited high BACE-1 inhibitions. However, these combinations preferred high ratios of stamen, since this sacred lotus part exhibited high BACE-1 inhibitory activities. BACE-1 inhibitors were previously reported as both peptidic and non-peptidic types. For peptidic inhibitors, intravenous administration of several peptides with specific amino acid sequences was reported to decrease brain amyloid-beta (Aβ) level in mouse models [48]. Non-peptidic BACE-1 inhibitors were previously reported to be small-sized compounds such as hydroxymethylcarbonyl (HMC) isostere, which is a substrate transition-state mimic in BACE-1 reaction [49], as well as chemotypes including aminohydantoin, aminooxazolines, and aminothiazolines that were designed based on interactions with the enzyme active site [50]. Phenolics have also been proven to act as BACE-1 inhibitors [51]. Naringenin was reported to inhibit BACE-1 activity with the IC_50_ value of 30.31 µM [43], while quercetin, a stronger BACE-1 inhibitor, exhibited the IC_50_ value of 5.4 µM [52]. Kaempferol (the IC_50_ value of 14.7 µM) and myricetin (the IC_50_ value of 2.8 µM) were also reported to effectively inhibit BACE-1 reaction [52]. According to our results, BACE-1 inhibitory activities of sacred lotus extracts might stem from peptidic inhibitors rather than non-peptidic inhibitors, since stamen, old leaf, and petal with high phenolic contents exhibited lower inhibitory activities than leaf stalk and flower stalk with lower phenolic contents.

Our findings suggested that an aqueous extract of stamen could be promoted and applied in functional food development for AD prevention. However, antagonist flavonoids-flavonoids interaction occurred between stamen, old leaf, and petal. Besides, the factors affecting the bioactive compounds crossing the blood brain barrier using cell culture technique and the effect of an aqueous extract on living organism (in vivo studies) should also be investigated. Further in-depth elucidation of these issues is required.

## 4. Materials and Methods

### 4.1. Sample Collection, Preparation, and Extraction

Dry sacred lotus (*Nelumbo nucifera*) parts including seed embryo, flower stalk, stamen, old leaf, petal, and leaf stalk were obtained from Kwan Phayao Lotus Community Enterprise, Phayao, Thailand during October 2018. The samples were ground into a fine powder using a grinder (Philips 600 W series from Philips Electronics Co., Ltd., Jakarta, Indonesia). The powder was packed in vacuum aluminum foil bags and kept at −20 °C in a freezer until required for further analysis. 

Color of the dry samples was analyzed using a ColorFlex EZ Spectrophotometer (Hunter Associates Laboratory, Reston, VA, USA) and expressed as CIELAB units, including L* representing dark (0) to white (100) colors, a* representing green (-) to red (+) colors and b* representing blue (-) to yellow (+) colors [53]. Moisture contents of the powdered samples were analyzed using a Halogen Moisture Analyzer (HE53 series from Mettler-Toledo AG, Greifensee, Switzerland) and presented as percentage moisture content (w/w) using the following equation:

(1) $$\%\;\mathrm{moisture}\;(w/w)=\frac{W_{w}\;-\;W_{d}}{W_{w}}\times \;100,$$

where *W_w_* and *W_d_* are weights of powdered samples before and after moisture evaporation, respectively. Colors and moisture contents are shown in Supplementary Table S1.

Optimization of extraction conditions was performed following the method of Sripum et al. 2017 [54]. Powdered samples were dissolved in distilled water using various solid-to-liquid ratios. The mixtures were then incubated in a temperature-controlled water bath shaker (WNE45 series from Memmert GmBh, WI, USA) at particular extraction temperatures with shaking for diverse time periods. The mixtures were then centrifuged at 3800× *g* using a Hettich^®^ Rotina 38R refrigerated centrifuge (Andreas Hettich GmbH, Tuttlingen, Germany) for 15 min. The supernatant was filtered through 54a 0.45 µM PES membrane syringe filter. All extracted samples were kept at −20 °C until required for further analyses.

To investigate the effect of solid-to-liquid ratio, powdered samples were prepared in deionized water at different concentrations (10, 20, 30, and 40 mg/mL), while fixing extraction temperature at 50 °C and shaking time for 2 h. The effect of shaking times (0.5, 1, 2, and 4 h) was investigated using a fixed extract concentration of 10 mg/mL with extraction temperature at 50 °C. Lastly, the effect of extraction temperatures (30, 50, 70, 90 °C) was examined using a fixed extract concentration at 10 mg/mL with shaking time of 1 h.

### 4.2. Determination of Antioxidant Activity

Antioxidant activities of the sacred lotus extracts were determined utilizing 2,2-diphenyl-1-picrylhydrazyl (DPPH) radical scavenging, ferric ion reducing antioxidant power (FRAP), and oxygen radical absorbance capacity (ORAC) assays, performed according to well-established protocols as previously described [55,56,57,58].

### 4.3. Determination of Total Phenolic Contents, Total Anthocyanin Contents (TACs), and Phenolic Profiles

Total phenolic contents (TPCs) of sacred lotus extracts were determined using Folin-Ciocalteu reagent [55,56,57,58]. Gallic acid (10–200 µg/mL) was used as a standard, with results reported as mg gallic acid equivalent (GAE)/g dried weight (DW).

Total anthocyanidin contents (TACs) of sacred lotus extracts were evaluated using the pH differential method as described elsewhere [59]. Cyanidin 3-*O*-glucoside (2–63 µg/mL) was used as a standard, and results were reported as mg cyanidin 3-*O*-glucoside equivalent (C3GE)/g DW.

High-performance liquid chromatography (HPLC) was performed to analyze phenolic profiles as described previously [60]. Briefly, dry sample (0.5 g) was dissolved in 62.5% (*v*/*v*) aqueous methanol containing 0.5 g/L tBHQ (40 mL) and 6 N HCl (10 mL) before incubating in an 80 °C temperature-controlled water bath shaker (WNE45 series from Memmert GmBh, Eagle, WI, USA) for 2 h. The mixture was sonicated in an ultrasonic cleansing bath (Branson Ultrasonics^TM^ M series, Branson Ultrasonics Corp., Danbury, CT, USA) for another 5 min before filtration through a 0.22 µM PTFE membrane syringe filter. The filtrate containing phenolics was subjected to an Agilent 1100 HPLC system with a photodiode array detector and a 5 µm Zorbax Eclipse XDB-C_18_ column (150 × 4.6 mm) (Agilent Technologies, Santa Clara, CA, USA). Gradient mobile phases consisted of Milli-Q water (18.2 MΩ.cm resistivity at 25 °C) containing 0.05% (*v*/*v*) TFA (solvent A), methanol containing 0.05% (*v*/*v*) TFA (solvent B) and acetonitrile containing 0.05% (*v*/*v*) TFA (solvent C) with a constant flow rate of 0.6 mL/min (Table 9). Detections at 280, 325, 338, and 368 nm were selected for visualization of the phenolics in sacred lotus extracts by comparing retention time (*t_R_*) and spectral fingerprints with the standards using ChemStation software (Agilent Technologies, Santa Clara, CA, USA). Standards for phenolic acids including 4-hydroxybenzoic acid (>99.0% GC, T), caffeic acid (>98.0% HPLC, T), chlorogenic acid (>98.0% HPLC, T), ferulic acid (>98.0% GC, T), *p*-coumaric acid (>98.0% GC, T), sinapic acid (>99.0% GC, T), and syringic acid (>97.0% T) were sourced from Tokyo Chemical Industry (Tokyo, Japan), while gallic acid (97.5–102.5% T) was purchased from Sigma-Aldrich (St. Louis, MO, USA). Standards for flavonoids including apigenin (>98.0% HPLC), hesperidin (>90.0% HPLC, T), kaempferol (>97.0% HPLC), luteolin (>98.0% HPLC), myricetin (>97.0% HPLC), naringenin (>93.0% HPLC, T), isorhamnetin (>95.0% HPLC), and quercetin (>98.0% HPLC, E) were purchased from Tokyo Chemical Industry (Tokyo, Japan), while isorhamnetin (≥99.0% HPLC) was bought from Extrasynthese (Genay, France).

Anthocyanidins were identified utilizing HPLC analysis as previously described [61,62]. The dry sample (500 mg) was mixed with 50% (*v*/*v*) aqueous methanol containing 2 N HCl (5 mL) before incubating at 100 ± 2 °C in a water bath (TW20 series from Julabo GmbH, Seelbach, Germany) for 1 h. The mixture was filtrated through a 0.22 µM PTFE membrane syringe filter, and the filtrate was subjected to an Ultimate 3000 HPLC system with diode array and multiple-wavelength detectors from Thermo Fisher Scientific, Dreieich, Germany with a 5 µm ReproSil-Pur^®^ ODS-3 column (250 × 4.6 mm) from Dr. Maisch GmbH (Ammerbuch, Germany). An isocratic mobile phase including Milli-Q water (18.2 MΩ.cm conductivity) containing 0.4% (*v*/*v*) TFA (solvent A) and acetonitrile containing 0.4% *v*/*v* TFA (solvent B) at a ratio of 82% solvent A and 18% solvent B with a constant flow rate of 1.0 mL/min was established as the HPLC conditions. Detection at 530 nm was selected for visualization of anthocyanidins in sacred lotus extracts by comparing *t_R_* and spectral fingerprints with the standards using Chromeleon^TM^ Chromatography Data System (CDS) software (Thermo Fisher Scientific, Dreieich, Germany). Standards for anthocyanidins including cyanidin (≥96.0% HPLC), delphinidin (≥97.0% HPLC), malvidin (≥97.0% HPLC), peonidin (≥97.0% HPLC), and petunidin (≥95.0% HPLC) were purchased from Extrasynthese (Genay, France).

Limit of detection (LOD), limit of quantitation (LOQ), and precision of HPLC standards were shown in Supplementary Table S2. The validation conditions of each parameter were carried based on the protocol of Srinuanchai et al. 2019 [63]. LOD and LOQ were determined from the linear calibration curve, which expressed as the following equation:

*y* = *a* + *bx* (2)

where *y* is an area under the peak, *x* is a standard concentration, *a* is a *y*-intercept, and *b* is a slope of the calibration curve. LOD and LOQ were expressed as the following equation:

LOD = 3.3*S_a_/b* (3)

LOQ = 10*S_a_*/*b* (4)

where *S_a_* is a standard deviation of the response (*y*-intercept), and *b* is a slope of the calibration curve. Intra-day precision was expressed as percentage of relative standard deviation (%RSD) and determined using the following equation:

%RSD = 100 × (*S_tR_*/*Mean_tR_*) (5)

where *S_tR_* is a standard deviation of the retention time, and *Mean_tR_* is the mean of the retention time measured at all concentrations of each standard.

### 4.4. Determination of Enzyme Inhibitory Activities

Inhibitory activities against acetylcholinesterases (AChE) and butyrylcholinesterase (BChE) were determined as previously described [60,64,65]. The AChE inhibitory assay was composed of 20 ng of *Electrophorus electricus* AChE (1000 units/mg, 100 μL) in 50 mM KPB (pH 7.0), 16 mM 5,5-dithio-*bis*-(2-nitrobenzoic acid) (DTNB, 10 μL), 0.8 mM acetylthiocholine (40 μL) in 50 mM KPB (pH 7.0), and sacred lotus extract (50 μL). The reaction was visualized for 1 h at a wavelength of 412 nm using a microplate reader (Synergy^TM^ HT 96-well UV-visible spectrophotometer with Gen5 data analysis software from BioTek Instruments, Inc., Winooski, VT, USA). The BChE inhibitory assay was similarly established, with utilization of 100 ng equine serum BChE (≥10 units/mg protein, 100 μL) in 50 mM KPB (pH 7.0) containing 1 mM MgCl_2_ and 0.1 mM butyrylthiocholine (40 μL) in 50 mM KPB (pH 7.0) as the enzyme and substrate, respectively. All enzymes, chemicals, and reagents in enzyme inhibitory assays were purchased from Sigma-Aldrich (St. Louis, MO, USA). Percentage inhibition was calculated as follows:

(6) $$\%\;\mathrm{inhibition}=\left(1-\;\frac{B-b}{A\;-\;a}\right)\;\times \;100,$$

where *A* is the initial velocity of the reaction with enzyme, *a* is the initial velocity of the reaction without enzyme, *B* is the initial velocity of the enzyme reaction with extract, and *b* is the initial velocity of the reaction with extract but without enzyme. The initial velocity of *a* and *b* are very close to zero; thus, these values can be negligible. Efficiency of sacred lotus extracts against the enzyme reaction was also determined using the half-maximal inhibitory concentration (IC_50_), analyzed by a dose-response plot of sacred lotus extracts versus percentage of inhibition.

Inhibitory activities against beta-secretase (BACE-1) were determined using a BACE-1 activity detection kit (Sigma-Aldrich, St. Louis, MO, USA), following the manufacturer’s instructions. Results were expressed as percentage of BACE-1 inhibition as mentioned above.

### 4.5. Statistical Analysis

All experiments were carried out in triplicate (*n* = 3) and expressed as mean ± standard deviation (SD). One-way analysis of variance (ANOVA) followed by Duncan’s multiple comparison test was performed to determine the significant differences between values with *p* < 0.05. 

## Figures and Tables

**Table 1:** Effects of different sacred lotus stamen extract concentrations regarding total phenolic contents (TPCs) and antioxidant activities determined by FRAP assay.

Independent Variable (Extract Concentration, mg/mL)	Dependent Variables		Controlled Variables
	TPCs (mg GAE/g DW)	Antioxidant Activities (µmol TE/g DW)	
10	56.52 ± 4.92 ^a^	731.80 ± 22.13 ^a^	Extraction temperature 50 °C Shaking time 2 h
20	52.98 ± 4.14 ^a^	656.81 ± 22.03 ^b^	
30	44.40 ± 2.32 ^b^	476.46 ± 19.66 ^c^	
40	38.27 ± 1.21 ^c^	420.85 ± 23.79 ^d^	
50	37.40 ± 2.10 ^c^	420.70 ± 9.90 ^d^	

Values are expressed as mean ± standard deviation (SD) of triplicate experiments (*n* = 3). GAE: gallic acid equivalent; TE: Trolox equivalent; DW: dry weight; different lower case letters in each column indicate significant differences at *p* < 0.05 calculated by one-way analysis of variance (ANOVA) and Duncan’s multiple comparison test.

**Table 2:** Effects of different shaking times on sacred lotus stamen extraction regarding total phenolic contents (TPCs) and antioxidant activities determined by FRAP assay.

Independent Variable (Shaking Time, Hour)	Dependent Variables		Controlled Variables
	TPCs (mg GAE/g DW)	Antioxidant Activities (µmol TE/g DW)	
0.5	62.09 ± 1.59 ^a^	655.02 ± 41.96 ^b^	Extraction temperature 50 °C Extract concentration 10 mg/mL
1.0	57.78 ± 5.69 ^ab^	722.98 ± 10.00 ^a^	
2.0	54.64 ± 5.21 ^b^	661.56 ± 25.72 ^b^	
4.0	59.50 ± 5.74 ^ab^	659.11 ± 14.09 ^b^	

Values are expressed as mean ± standard deviation (SD) of triplicate experiments (*n* = 3). GAE: gallic acid equivalent; TE: Trolox equivalent; DW: dry weight; different lower case letters in each column indicate significant differences at *p* < 0.05 calculated by one-way analysis of variance (ANOVA) and Duncan’s multiple comparison test.

**Table 3:** Effects of different temperatures on sacred lotus stamen extraction regarding total phenolic contents (TPCs) and antioxidant activities determined by FRAP assay.

Independent Variable (Extraction Temperature, °C)	Dependent Variables		Controlled Variables
	TPCs (mg GAE/g DW)	Antioxidant Activities (µmol TE/g DW)	
30	51.19 ± 4.01 ^b^	655.57 ± 43.50 ^c^	Shaking time 1 h Extract concentration 10 mg/mL
50	51.98 ± 5.09 ^b^	646.04 ± 57.57 ^bc^	
70	60.17 ± 5.04 ^a^	697.49 ± 53.88 ^ab^	
90	64.27 ± 4.27 ^a^	742.75 ± 50.83 ^a^	

Values are expressed as mean ± standard deviation (SD) of triplicate experiments (*n* = 3). GAE: gallic acid equivalent; TE: Trolox equivalent; DW: dry weight; different lower case letters in each column indicate significant differences at *p* < 0.05 calculated by one-way analysis of variance (ANOVA) and Duncan’s multiple comparison test.

**Table 4:** Quantification of phenolics, total phenolic contents (TPCs), and total anthocyanin contents (TACs) in different parts of sacred lotus (*Nelumbo nucifera*).

Phenolic Contents	Parts of Sacred Lotus					
	Seed Embryo	Flower Stalk	Stamen	Old Leaf	Petal	Leaf Stalk
**Phenolic acids (mg/100 g DW)**						
**Gallic acid**	ND	ND	ND	49.38 ± 4.83 ^c^	277.84 ± 6.36 ^a^	163.09 ± 8.58 ^b^
***p*** **-Coumaric acid**	105.34 ± 2.93 ^a^	ND	10.78 ± 0.38 ^b^	ND	ND	ND
**Ferulic acid**	24.71 ± 2.03	ND	ND	ND	ND	ND
**Flavonoids** **(mg/100 g DW)**						
**Myricetin**	ND	8.89 ± 0.83 ^a^	7.63 ± 0.35 ^b^	ND	8.55 ± 0.29 ^a^^b^	ND
**Luteolin**	37.50 ± 1.87 ^a^	4.89 ± 0.35 ^c^	ND	ND	ND	12.43 ± 0.77 ^b^
**Quercetin**	81.79 ± 3.57 ^c^	59.91 ± 5.64 ^d^	43.94 ± 2.08 ^d^	458.56 ± 33.45 ^a^	196.34 ± 19.03 ^b^	35.95 ± 1.94 ^d^
**Naringenin**	2241.51 ± 18.41 ^a^	2213.41 ± 11.35 ^a^	2185.84 ± 24.21 ^a^	1064.17 ± 75.38 ^c^	2226.69 ± 13.66 ^a^	1918.10 ± 37.81 ^b^
**Kaempferol**	4.92 ± 0.41 ^c^	6.40 ± 0.64 ^c^	160.71 ± 13.66 ^b^	3.87 ± 0.31 ^c^	197.83 ± 19.81 ^a^	ND
**Isorhamnetin**	11.56 ± 0.85 ^c^	3.51 ± 0.28 ^c^	192.09 ± 15.70 ^b^	2.67 ± 0.09 ^c^	237.85 ± 13.86 ^a^	6.80 ± 0.35 ^c^
**Cyanidin**	1901.52 ± 14.15 ^a^	12.02 ± 0.09 ^e^	115.79 ± 10.21 ^d^	184.82 ± 11.38 ^c^	349.98 ± 24.28 ^b^	7.15 ± 0.74 ^e^
**Delphinidin**	691.58 ± 9.84 ^b^	20.70 ± 0.24 ^d^	211.63 ± 17.21 ^c^	39.46 ± 2.42 ^d^	1837.27 ± 52.67 ^a^	6.15 ± 1.05 ^d^
**Total phenolic contents** **(mg GAE/g DW)**	12.84 ± 0.22 ^c^	4.33 ± 0.11 ^d^	36.37 ± 0.73 ^b^	39.09 ± 0.79 ^a^	12.25 ± 0.36 ^c^	2.72 ± 0.10 ^e^
**Total anthocyanin contents** **(mg C3GE/g DW)**	ND	ND	0.23 ± 0.02 ^a^	ND	0.05 ± 0.00 ^b^	ND

All data are expressed as mean ± standard deviation (SD) of triplicate experiments (*n* = 3). GAE: gallic acid equivalent; C3GE: cyanidin 3-*O*-glucoside equivalent; DW: dry weight; ND: not detected; different lower case letters in each row indicate significant differences at *p* < 0.05 calculated by one-way analysis of variance (ANOVA) and Duncan’s multiple comparison test.

**Table 5:** Antioxidant activities for different parts of sacred lotus (*Nelumbo nucifera*).

Sacred Lotus Parts	Antioxidant Activities		
	DPPH Radical Scavenging Assay (µmol TE/100 g DW)	FRAP Assay (µmol TE/g DW)	ORAC Assay (µmol TE/g DW)
Seed embryo	0.71 ± 0.04 ^c^	81.81 ± 1.53 ^c^	292.81 ± 7.33 ^d^
Flower stalk	0.63 ± 0.04 ^d^	22.98 ± 0.39 ^e^	214.60 ± 19.31 ^e^
Stamen	1.06 ± 0.01 ^b^	281.46 ± 4.12 ^a^	561.98 ± 16.44 ^b^
Old leaf	1.21 ± 0.00 ^a^	231.08 ± 1.70 ^b^	1001.12 ± 58.33 ^a^
Petal	0.86 ± 0.06 ^c^	71.65 ± 2.71 ^d^	370.49 ± 16.82 ^c^
Leaf stalk	0.43 ± 0.01 ^e^	15.39 ± 0.38 ^f^	119.94 ± 3.26 ^f^

All data are expressed as mean ± standard deviation (SD) of triplicate experiments (*n* = 3). TE: Trolox equivalent; DW: dry weight; different lower case letters indicate significant differences (*p* < 0.05) of antioxidant activities in the same column using one-way analysis of variance (ANOVA) and Duncan’s multiple comparison test.

**Table 6:** Antioxidant activities and total phenolic contents (TPCs) of different mixtures (ratios) of stamen, old leaf, and petal of sacred lotus (*Nelumbo nucifera*).

Number of Recipe	Ratio			TPCs (mg GAE/g DW)	Antioxidant Activities		
	Stamen	Old Leaf	Petal		DPPH Radical Scavenging Assay (µmol TE/100 g DW)	FRAP Assay (µmol TE/g DW)	ORAC Assay (µmol TE/g DW)
1	3	0	0	38.55 ± 3.58 ^b^	1.12 ± 0.03 ^a^	296.50 ± 24.11 ^a^	327.05 ± 25.12 ^hij^
2	0	3	0	43.44 ± 1.73 ^a^	1.09 ± 0.06 ^ab^	143.09 ± 13.01 ^efg^	436.14 ± 37.03 ^e^
3	0	0	3	6.73 ± 0.43 ^m^	0.76 ± 0.03 ^g^	83.68 ± 4.95 ^lm^	212.26 ± 20.66 ^l^
4	1	1	1	29.27 ± 1.52 ^c^	0.99 ± 0.08 ^cdefg^	190.12 ± 10.82 ^b^	389.63 ± 32.88 ^f^
5	1	1	2	24.58 ± 0.99 ^f^	0.92 ± 0.08 ^defg^	91.56 ± 8.60 ^kl^	378.60 ± 28.76 ^fg^
6	1	1	3	16.73 ± 0.86 ^hi^	0.81 ± 0.08 ^defg^	100.46 ± 9.85 ^jk^	293.28 ± 27.93 ^jk^
7	1	2	1	19.03 ± 1.33 ^g^	0.90 ± 0.05 ^cdefg^	65.46 ± 4.36 ^n^	443.74 ± 42.47 ^de^
8	1	2	2	7.62 ± 0.40 ^m^	0.90 ± 0.07 ^cdefg^	120.49 ± 5.33 ^i^	565.31 ± 53.97 ^bc^
9	1	2	3	10.61 ± 0.83 ^l^	0.87 ± 0.08 ^cdefg^	174.74 ± 17.10 ^c^	272.60 ± 17.93 ^k^
10	1	3	1	27.97 ± 2.55 ^cd^	0.95 ± 0.09 ^bcdef^	144.00 ± 13.01 ^efg^	289.13 ± 13.12 ^jk^
11	1	3	2	15.50 ± 1.25 ^ij^	0.92 ± 0.07 ^cdefg^	135.64 ± 5.41 ^gh^	487.31 ± 39.40 ^d^
12	1	3	3	13.32 ± 0.84 ^k^	0.94 ± 0.04 ^bcdef^	145.43 ± 7.08 ^efg^	670.83 ± 66.86 ^a^
13	2	1	1	16.47 ± 0.42 ^i^	0.89 ± 0.07 ^efg^	153.19 ± 7.59 ^de^	449.00 ± 34.81 ^de^
14	2	1	2	21.07 ± 2.07 ^gh^	0.90 ± 0.08 ^cdefg^	144.52 ± 4.37 ^efg^	361.55 ± 24.43 ^fgh^
15	2	1	3	18.59 ± 1.46 ^gh^	0.87 ± 0.09 ^cdefg^	126.87 ± 7.79 ^hi^	335.16 ± 23.65 ^ghij^
16	2	2	1	26.91 ± 2.49 ^de^	0.99 ± 0.06 ^abc^	120.37 ± 7.85 ^i^	603.10 ± 30.39 ^b^
17	2	2	3	20.70 ± 1.56 ^g^	0.89 ± 0.07 ^cdefg^	54.58 ± 5.10 ^o^	479.82 ± 36.24 ^de^
18	2	3	1	29.77 ± 1.71 ^c^	0.97 ± 0.07 ^abcd^	176.15 ± 17.33 ^c^	468.54 ± 36.90 ^de^
19	2	3	2	18.83 ± 1.46 ^gh^	0.95 ± 0.09 ^bcde^	177.92 ± 7.78 ^c^	441.32 ± 43.87 ^de^
20	2	3	3	25.86 ± 2.25 ^def^	0.97 ± 0.09 ^abcd^	141.13 ± 6.81 ^fg^	376.71 ± 35.28 ^fg^
21	3	1	1	25.05 ± 2.21 ^ef^	0.91 ± 0.05 ^cdefg^	122.62 ± 7.61 ^i^	538.62 ± 35.09 ^c^
22	3	1	2	13.38 ± 0.93 ^k^	0.78 ± 0.04 ^fg^	162.09 ± 9.82 ^d^	364.32 ± 36.17 ^fgh^
23	3	1	3	13.73 ± 0.82 ^jk^	0.84 ± 0.07 ^g^	147.46 ± 9.15 ^ef^	352.98 ± 32.49 ^fgh^
24	3	2	1	27.88 ± 1.00 ^cd^	0.95 ± 0.06 ^bcde^	179.46 ± 10.81 ^c^	303.70 ± 24.26 ^ijk^
25	3	2	2	26.38 ± 2.51 ^def^	0.91 ± 0.06 ^cdefg^	199.39 ± 14.98 ^b^	437.06 ± 35.96 ^e^
26	3	2	3	14.94 ± 0.65 ^ijk^	0.87 ± 0.07 ^cdefg^	75.77 ± 5.78 ^m^	220.92 ± 15.99 ^l^
27	3	3	1	19.16 ± 1.39 ^g^	1.03 ± 0.09 ^abc^	105.56 ± 6.24 ^j^	341.19 ± 22.18 ^ghi^
28	3	3	2	18.69 ± 0.47 ^gh^	0.91 ± 0.09 ^defg^	159.00 ± 11.66 ^d^	326.47 ± 28.82 ^hij^

All data were expressed as mean ± standard deviation (SD) of triplicate experiments (*n* = 3). GAE: gallic acid equivalent; TE: trolox equivalent; DW: dry weight; different lower case letters indicate significant differences (*p* < 0.05) of either total phenolic contents or antioxidant activities in the same column using one-way analysis of variance (ANOVA) and Duncan’s multiple comparison test.

**Table 7:** Inhibitory activities against acetylcholinesterase (AChE), butyrylcholinesterase (BChE) and beta-secretase (BACE-1) of sacred lotus (*Nelumbo nucifera*) extracts.

Parts of Sacred Lotus	Enzyme Inhibitory Activity				
	AChE		BChE		BACE-1
	% Inhibition *	IC_50_ (mg/mL)	% Inhibition *	IC_50_ (mg/mL)	% Inhibition *
Seed embryo	81.38 ± 0.82 ^d^	4.23 ± 0.69 ^b^	80.21 ± 1.27 ^c^	10.80 ± 1.31 ^d^	44.51 ± 3.39 ^d^
Flower stalk	83.67 ± 1.11 ^c^	3.90 ± 0.41 ^b^	58.47 ± 4.03 ^d^	11.94 ± 1.01 ^d^	75.61 ± 1.22 ^a^
Stamen	89.46 ± 0.51 ^ab^	0.66 ± 0.03 ^a^	91.61 ± 0.51 ^a^^b^	0.08 ± 0.00 ^a^	70.73 ± 1.06 ^b^
Old leaf	90.70 ± 1.18 ^a^	1.09 ± 0.08 ^a^	93.72 ± 2.66 ^a^	2.16 ± 0.02 ^b^	54.68 ± 2.75 ^c^
Petal	87.89 ± 0.85 ^b^	3.78 ± 0.32 ^b^	88.75 ± 0.62 ^b^	6.11 ± 0.33 ^c^	51.83 ± 1.06 ^c^
Leaf stalk	55.75 ± 1.40 ^e^	9.47 ± 0.80 ^c^	34.72 ± 1.70 ^e^	11.87 ± 0.74 ^d^	74.80 ± 2.82 ^a^

All data are expressed as mean ± standard deviation (SD) of triplicate experiments (*n* = 3). Different lower case letters indicate significant differences (*p* < 0.05) of the enzyme inhibitory activities in the same column using one-way analysis of variance (ANOVA) and Duncan’s multiple comparison test; * extract concentration = 10 mg/mL.

**Table 8:** Inhibitory activities against acetylcholinesterase (AChE), butyrylcholinesterase (BChE) and beta-secretase (BACE-1) of different mixtures (ratios) of stamen, old leaf, and petal of sacred lotus (*Nelumbo nucifera*).

Number of Recipe	Ratio			% Inhibition *		
	Stamen	Old Leaf	Petal	AChE	BChE	BACE-1
1	3	0	0	80.58 ± 4.70 ^a^	99.30 ± 5.12 ^a^	50.26 ± 1.18 ^i^
2	0	3	0	76.14 ± 6.46 ^a^	30.61 ± 2.68 ^k^	62.52 ± 0.78 ^bcd^
3	0	0	3	29.43 ± 2.37 ^hij^	22.35 ± 1.14 ^no^	44.29 ± 1.35 ^j^
4	1	1	1	52.84 ± 3.24 ^bc^	41.59 ± 3.62 ^hi^	58.29 ± 1.29 ^cdef^
5	1	1	2	48.48 ± 2.49 ^bcd^	34.39 ± 3.04 ^j^	58.09 ± 1.84 ^defg^
6	1	1	3	35.53 ± 2.94 ^fghi^	44.09 ± 3.66 ^gh^	61.84 ± 3.12 ^bcd^
7	1	2	1	50.05 ± 2.61 ^bcd^	22.40 ± 1.91 ^no^	60.99 ± 2.81 ^cde^
8	1	2	2	46.11 ± 6.22 ^cde^	21.30 ± 1.63 ^o^	58.09 ± 1.84 ^defg^
9	1	2	3	15.25 ± 1.46 ^l^	23.54 ± 1.77 ^mno^	54.68 ± 1.56 ^fghi^
10	1	3	1	22.86 ± 2.23 ^kl^	28.60 ± 2.18 ^kl^	63.88 ± 3.90 ^abc^
11	1	3	2	13.66 ± 1.37 ^l^	17.36 ± 1.33 ^p^	56.39 ± 4.13 ^efgh^
12	1	3	3	18.20 ± 1.37 ^l^	25.59 ± 2.40 ^lmn^	55.37 ± 2.52 ^fghi^
13	2	1	1	55.09 ± 5.03 ^bcd^	65.33 ± 5.23 ^d^	58.09 ± 2.70 ^defg^
14	2	1	2	55.93 ± 4.37 ^bcd^	53.71 ± 3.08 ^ef^	53.49 ± 2.23 ^ghi^
15	2	1	3	29.41 ± 2.23 ^hij^	30.12 ± 2.67 ^k^	51.68 ± 2.19 ^hi^
16	2	2	1	35.98 ± 1.97 ^jk^	53.07 ± 4.55 ^f^	66.95 ± 3.35 ^ab^
17	2	2	3	19.29 ± 1.77 ^l^	37.08 ± 3.42 ^j^	61.91 ± 2.77 ^bcd^
18	2	3	1	28.74 ± 2.79 ^hij^	30.40 ± 2.62 ^k^	68.12 ± 1.05 ^a^
19	2	3	2	25.23 ± 1.65 ^ijk^	35.76 ± 2.73 ^j^	62.92 ± 4.34 ^abcd^
20	2	3	3	26.64 ± 2.37 ^hij^	27.45 ± 2.38 ^klm^	61.91 ± 2.77 ^bcd^
21	3	1	1	58.48 ± 3.19 ^b^	81.13 ± 1.59 ^b^	54.19 ± 3.52 ^fghi^
22	3	1	2	58.48 ± 3.19 ^b^	73.03 ± 5.83 ^c^	57.72 ± 1.74 ^defg^
23	3	1	3	49.39 ± 1.70 ^ghij^	57.73 ± 4.42 ^e^	54.70 ± 2.52 ^fghi^
24	3	2	1	49.15 ± 4.82 ^cdef^	61.71 ± 5.94 ^d^	62.92 ± 2.77 ^abcd^
25	3	2	2	41.74 ± 3.34 ^defg^	56.44 ± 4.18 ^ef^	62.75 ± 3.63 ^bcd^
26	3	2	3	37.15 ± 3.15 ^efgh^	38.39 ± 3.30 ^ij^	63.09 ± 4.86 ^abcd^
27	3	3	1	49.88 ± 2.98 ^bcd^	56.93 ± 4.95 ^ef^	62.92 ± 2.03 ^abcd^
28	3	3	2	31.02 ± 2.73 ^hij^	47.71 ± 3.23 ^g^	66.44 ± 3.03 ^ab^

All data are expressed as mean ± standard deviation (SD) of triplicate experiments (*n* = 3). Different lower case letters indicate significant differences (*p* < 0.05) of the enzyme inhibitory activities in the same column using one-way analysis of variance (ANOVA) and Duncan’s multiple comparison test; * extract concentrations for AChE and BChE inhibitory assays = 0.9 mg/mL, while BACE-1 inhibitory assay = 9 mg/mL.

**Table 9:** Solvent system of high-performance liquid chromatography (HPLC) to identify phenolic acids and flavonoids in sacred lotus extracts.

Time (min)	Flow Rate (mL/min)	Solvent A (%)	Solvent B (%)	Solvent C (%)
0	0.6	90	6	4
5	0.6	85	9	6
30	0.6	71	17.4	11.6
60	0.6	0	85	15
61	0.6	90	6	4
66	0.6	90	6	4

Solvent A = Milli-Q water containing 0.05% (*v*/*v*) TFA; solvent B = methanol containing 0.05% (*v*/*v*) TFA; solvent C = acetonitrile containing 0.05% (*v*/*v*) TFA.

## Supplementary Materials

The following are available online. Supplementary Table S1: Color (where L* describes darkness (−) to lightness (+), a* describes green (−) to red (+), and b* describes indigo (−) to yellow (+)) and percentage (%) of moisture content of sacred lotus samples.; Supplementary Table S2: The validation parameters of sacred lotus extract detection using HPLC analysis.; Supplementary Figure S1: High-performance liquid chromatograms of (A.) gallic acid, (B.) naringenin and sacred lotus extracts including (C.) seed embryo, (D.) flower stalk, (E.) stamen, (F.) old leaf, (G.) petal, and (H.) leaf stalk. Retention times (Rt) of phenolics in sacred lotus extracts are indicated at a wavelength of 280 nm.; Supplementary Figure S2: High-performance liquid chromatograms of (A.) p-coumaric acid, (B.) ferulic acid and sacred lotus extracts including (C.) seed embryo, (D.) flower stalk, (E.) stamen, (F.) old leaf, (G.) petal, and (H.) leaf stalk. Retention times (Rt) of phenolics in sacred lotus extracts are indicated at a wavelength of 325 nm.; Supplementary Figure S3: High-performance liquid chromatograms of (A.) luteolin, and sacred lotus extracts including (B.) seed embryo, (C.) flower stalk, (D.) stamen, (E.) old leaf, (F.) petal, and (G.) leaf stalk. Retention times (Rt) of phenolics in sacred lotus extracts are indicated at a wavelength of 338 nm.; Supplementary Figure S4: High-performance liquid chromatograms of (A.) myricetin, (B.) quercetin, (C.) kaempferol, (D.) isorhamnetin and sacred lotus extracts including (E.) seed embryo, (F.) flower stalk, (G.) stamen, (H.) old leaf, (I.) petal, and (J.) leaf stalk. Retention times (Rt) of phenolics in sacred lotus extracts are indicated at a wavelength of 368 nm.; Supplementary Figure S5: High-performance liquid chromatograms of (A.) cyanidin, (B.) delphinidin and sacred lotus extracts including (C.) seed embryo, (D.) flower stalk, (E.) stamen, (F.) old leaf, (G.) petal, and (H.) leaf stalk. Retention times (Rt) of phenolics in sacred lotus extracts are indicated at a wavelength of 530 nm.; Supplementary Figure S6: Scheme showed the IC50 plots against acetylcholinesterase (AChE) of sacred lotus extracts including (A.) seed embryo, (B.) flower stalk, (C.) stamen, (D.) old leaf, (E.) petal, and (F.) leaf stalk.; Supplementary Figure S7: Scheme showing the IC50 plots against butyrylcholinesterase (BChE) of sacred lotus extracts including (A.) seed embryo, (B.) flower stalk, (C.) stamen, (D.) old leaf, (E.) petal, and (F.) leaf stalk.
