# Supplementary material for: The Effect of Sacred Lotus (Nelumbo nucifera) and Its Mixtures on Phenolic Profiles, Antioxidant Activities, and Inhibitions of the Key Enzymes Relevant to Alzheimer’s Disease
Source: Molecules. 2020 Aug 14;25(16):3713. doi: 10.3390/molecules25163713 (PMC7463813; doi:10.3390/molecules25163713)

molecules

Supplementary materials

The effect of sacred lotus (*Nelumbo nucifera*) and its mixtures on phenolic profiles, antioxidant activities, and inhibitions of the key enzymes relevant to Alzheimer's disease

Piya Temviriyanukul [1,2], Varittha Sritalahareuthai [1], Natnicha Promyos [1], Sirinapa Thangsiri [1], Kanchana Pruesapan [3], Wanwisa Srinuanchai [4], Onanong Nuchuchua [4], Dalad Siriwan [5], Nattira On-nom [1,2] and Uthaiwan Suttisansanee [1,2,*]

[1] Institute of Nutrition, Mahidol University, Salaya, Phuttamonthon, Nakhon Pathom 73170, Thailand; piya.tem@mahidol.ac.th (P.T.); varittha.sri@hotmail.com (V.S.); natnicha.prm@mahidol.ac.th (N.P.); poo.sweet@hotmail.com (S.T.); nattira.onn@mahidol.ac.th (N.O.); uthaiwan.sut@mahidol.ac.th (U.S.)
[2] Food and Nutrition Academic and Research Cluster, Institute of Nutrition, Mahidol University, Salaya, Phuttamonthon, Nakhon Pathom 73170, Thailand
[3] Plant Varieties Protection Division, Department of Agriculture, Ministry of Agriculture and Cooperatives, Bangkok, Thailand, 10900; kpruesapan@gmail.com (K.P.)
[4] National Nanotechnology Center (NANOTEC), National Science and Technology Development Agency (NSTDA), Klong Luang, Pathum Thani, 12120, Thailand; wanwisa.sri@nanotec.or.th (W.S.); onanong@nanotec.or.th (O.N.)
[5] Institute of Food Research and Product Development, Kasetsart University, Chatuchak, Bangkok 10900, Thailand; dalad.s@ku.th
* Correspondence: uthaiwan.sut@mahidol.ac.th; Tel.: +662-800-2380 ext. 422

Supplementary Table S1:

Color (where L* describes darkness (−) to lightness (+), a* describes green (−) to red (+), and b* describes indigo (−) to yellow (+)) and percentage (%) of moisture content of sacred lotus samples.

Parts of sacred lotus	Color			Moisture content
	L*	a*	b*	(%)
Seed embryo	20.55 ± 0.01	2.65 ± 0.04	7.24 ± 0.05	12.10 ± 0.12
Flower stalk	22.68 ± 0.03	4.54 ± 0.01	7.78 ± 0.05	9.85 ± 0.50
Stamen	20.57 ± 0.06	2.24 ± 0.02	7.11 ± 0.03	8.10 ± 0.24
Old leaf	22.28 ± 0.04	(-)1.3 ± 0.03	10.42 ± 0.16	5.11 ± 0.11
Petal	24.57 ± 0.01	2.49 ± 0.02	9.58 ± 0.03	4.57 ± 0.25
Leaf stalk	24.20 ± 0.02	4.91 ± 0.03	12.31 ± 0.03	8.53 ± 0.28

All data were expressed as mean ± standard deviation (SD) of triplicate experiments (n = 3).

Supplementary Table S2:

The validation parameters of sacred lotus extract detection using HPLC analysis.

Standards	Linear range (µg/mL)	Linear regression equation	Correlation coefficient (R^2)	LOQ (µg/mL)	LOQ (µg/mL)	RSD (%)
**Phenolic acids**						
Gallic acid	0.78-200.00	y = 38.814x + 43.836	0.9998	2.66	8.07	1.37
4-Hydroxybenzoic acid	0.39-100.00	y = 54.999x - 11.469	0.9994	0.32	0.98	1.86
Caffeic acid	0.39-100.00	y = 76.614x + 15.797	0.9998	0.59	1.78	1.70
Chlorogenic acid	0.39-100.00	y = 45.107x - 8.1659	1.0000	0.03	0.10	1.59
Ferulic acid	0.56-142.86	y = 42.477x - 16.212	0.9990	0.49	1.48	1.58
p-Coumaric acid	0.56-142.86	y = 44.809x - 22.611	0.9991	0.57	1.73	1.97
Sinapic acid	0.39-100.00	y = 63.807x + 10.841	1.0000	0.22	0.68	1.01
Syringic acid	0.65-166.67	y = 37.286x - 24.557	0.9990	1.12	3.41	1.63
**Flavonoids**						
Apigenin	0.39-200.00	y = 40.778x + 34.994	0.9999	2.22	6.73	0.44
Hesperidin	0.65-333.33	y = 24.424x + 76.057	0.9990	2.56	7.77	0.64
Kaempferol	0.65-166.67	y = 47.952x + 43.855	0.9999	0.14	0.42	0.12
Luteolin	0.65-166.67	y = 44.717x + 28.899	0.9999	1.19	3.62	0.52
Myricetin	0.65-166.67	y = 43.048x + 16.879	0.9999	0.37	1.12	0.32
Naringenin	0.65-333.33	y = 30.205x + 46.292	0.9999	0.46	1.38	0.45
Quercetin	0.65-166.67	y = 46.975x + 38.753	0.9999	0.19	0.58	0.10
Isorhamnetin	1.30-333.33	y = 16.302x + 46.483	0.9992	0.45	1.37	0.39
**Anthocyanidins**						
Cyanidin	2.58-330.00	y = 1.5641x + 4.4497	0.9995	0.05	0.15	0.58
Delphinidin	2.58-330.00	y = 1.6280x + 5.0454	0.9992	0.77	2.34	0.45
Peonidin	2.58-330.00	y = 1.8154x + 4.1796	0.9995	3.08	9.34	0.73
Petunidin	3.91-125.00	y = 1.2904x + 4.4487	0.9951	0.78	2.36	0.28
Malvidin	3.91-125.00	y = 0.6093x + 4.1012	0.9945	1.82	5.53	0.24

Supplementary Figure 1:

High-performance liquid chromatograms of (A.) gallic acid, (B.) naringenin and sacred lotus extracts including (C.) seed embryo, (D.) flower stalk, (E.) stamen, (F.) old leaf, (G.) petal, and (H.) leaf stalk. Retention times (R_t) of phenolics in sacred lotus extracts are indicated at a wavelength of 280 nm.

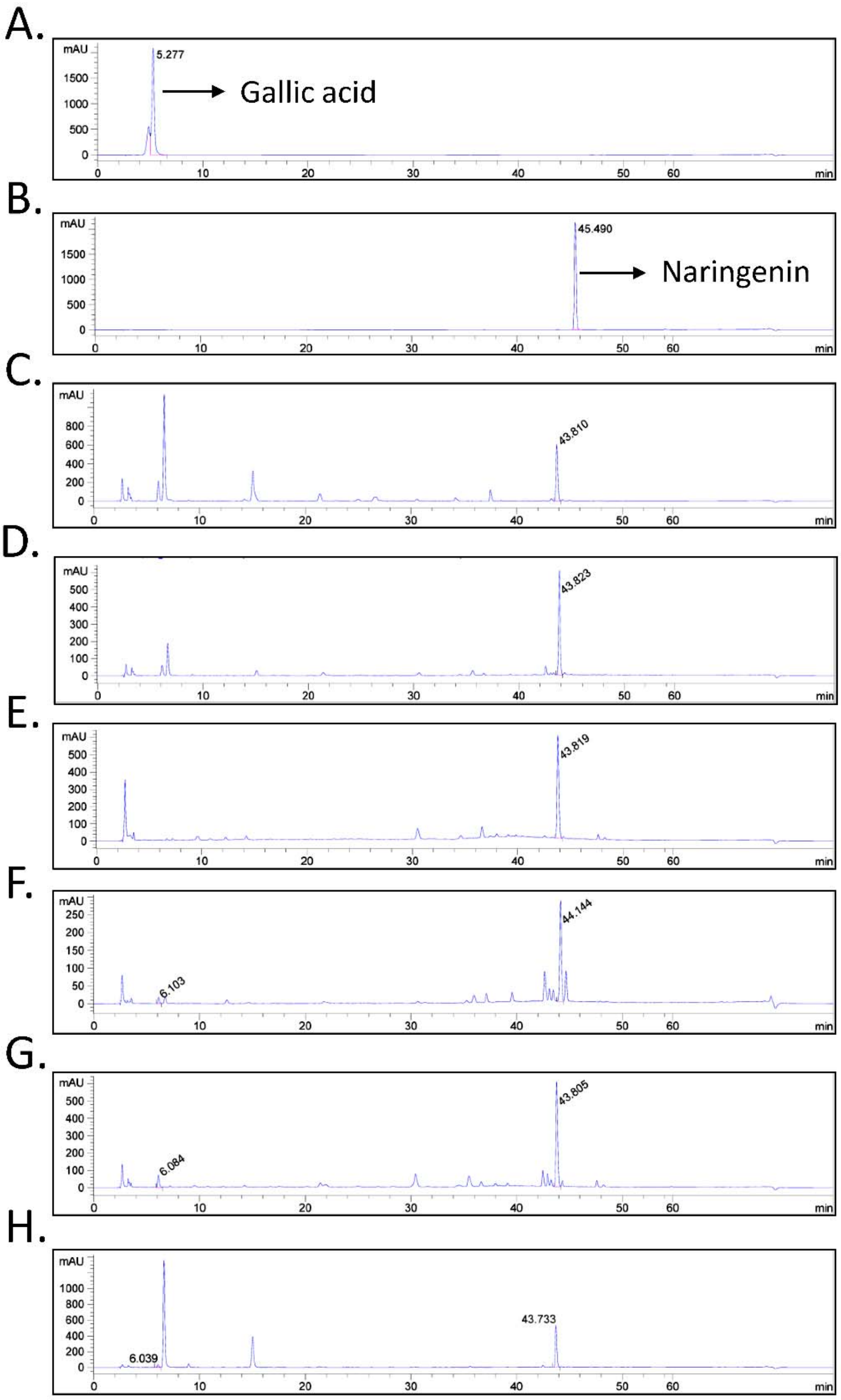

Supplementary Figure S2:

High-performance liquid chromatograms of (A.) *p*-coumaric acid, (B.) ferulic acid and sacred lotus extracts including (C.) seed embryo, (D.) flower stalk, (E.) stamen, (F.) old leaf, (G.) petal, and (H.) leaf stalk. Retention times (R_t) of phenolics in sacred lotus extracts are indicated at a wavelength of 325 nm

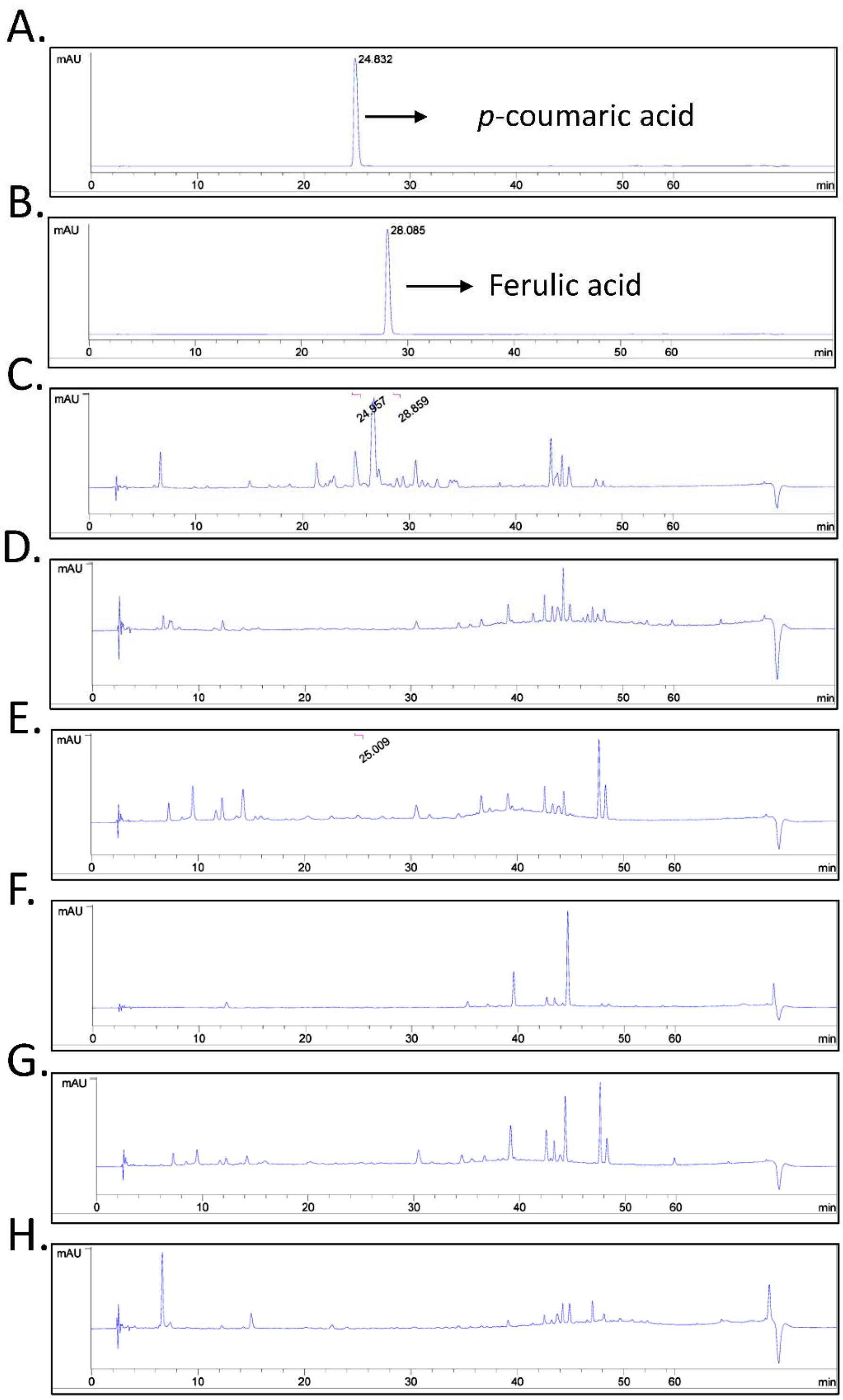

Supplementary Figure S3:

High-performance liquid chromatograms of (A.) luteolin, and sacred lotus extracts including (B.) seed embryo, (C.) flower stalk, (D.) stamen, (E.) old leaf, (F.) petal, and (G.) leaf stalk. Retention times (R_t) of phenolics in sacred lotus extracts are indicated at a wavelength of 338 nm.

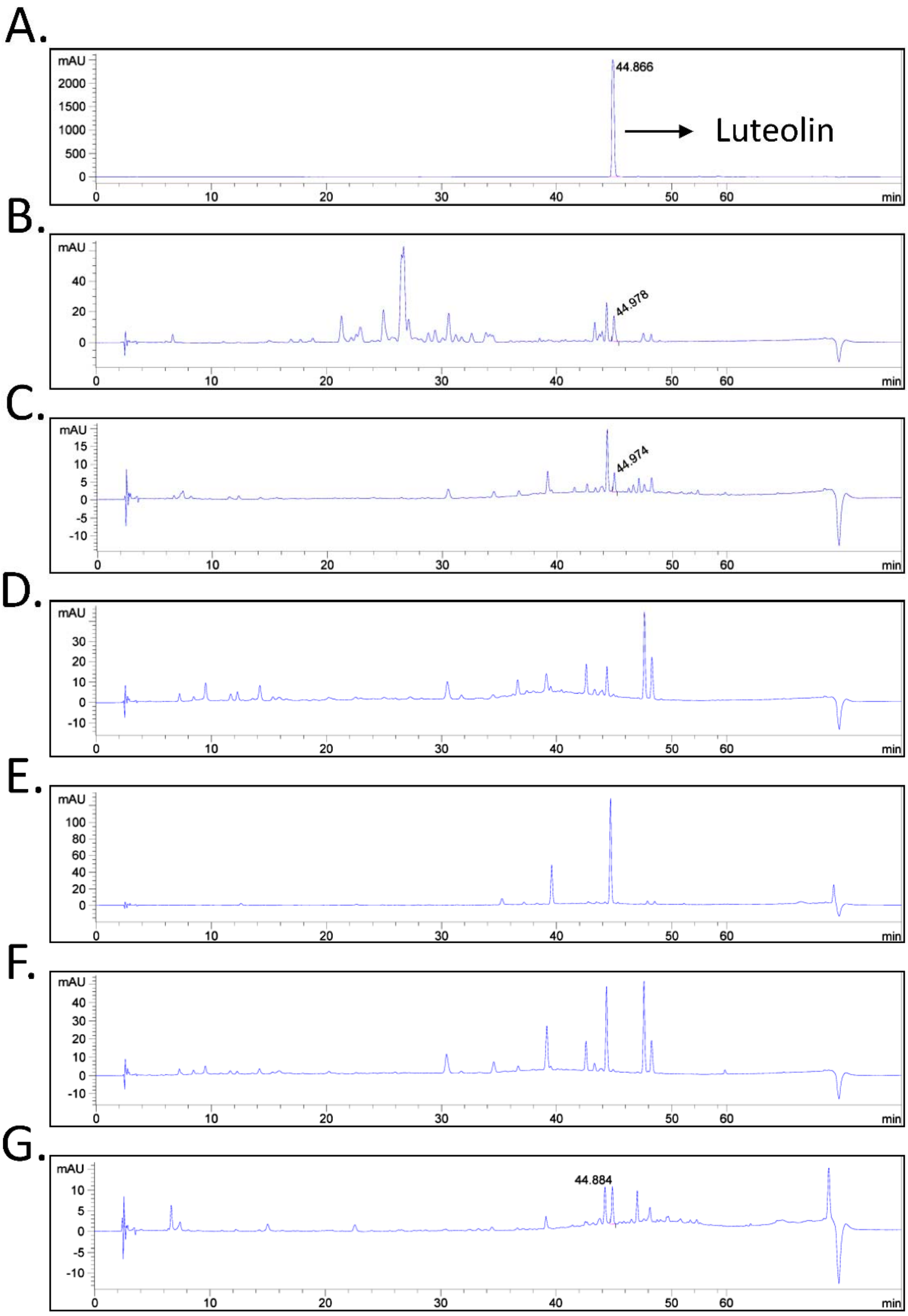

Supplementary Figure S4:

High-performance liquid chromatograms of (A.) myricetin, (B.) quercetin, (C.) kaempferol, (D.) isorhamnetin and sacred lotus extracts including (E.) seed embryo, (F.) flower stalk, (G.) stamen, (H.) old leaf, (I.) petal, and (J.) leaf stalk. Retention times (R_t) of phenolics in sacred lotus extracts are indicated at a wavelength of 368 nm.

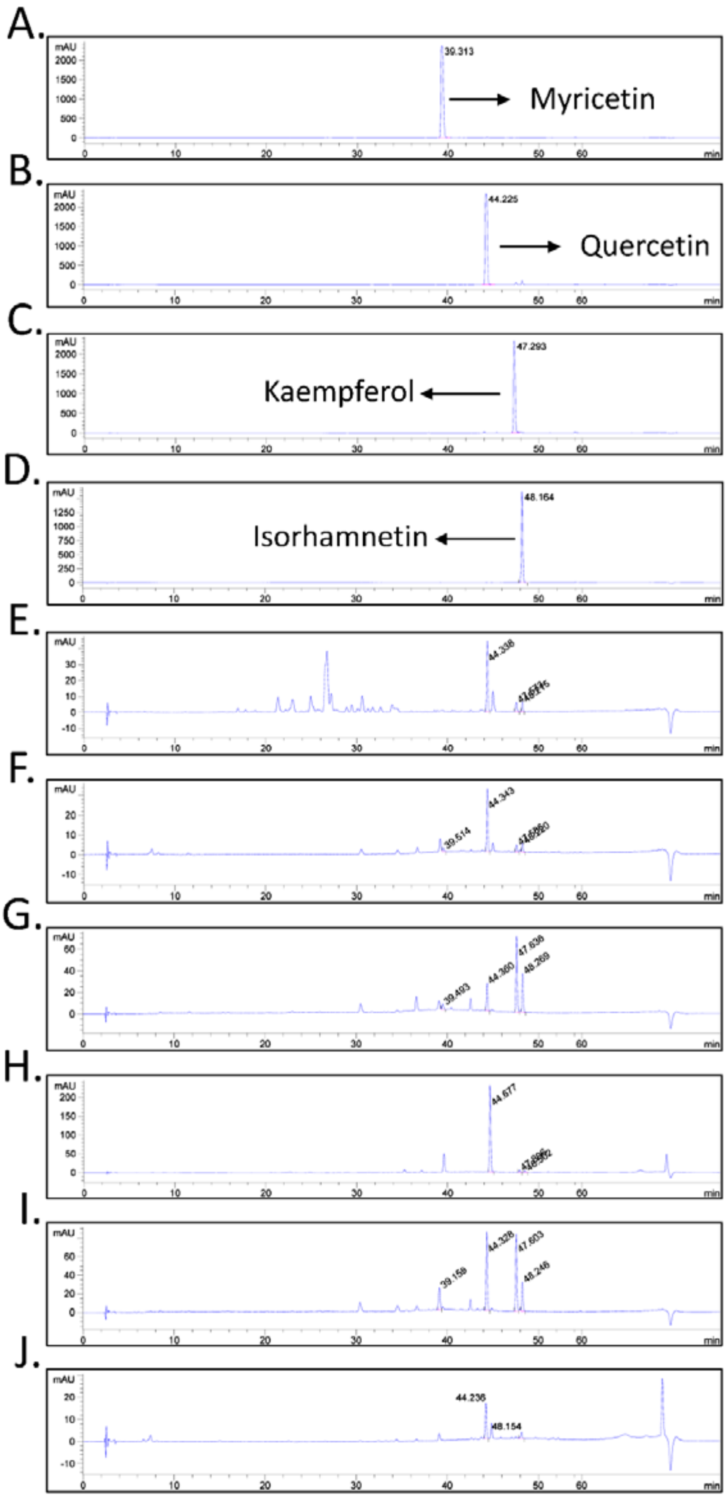

Supplementary Figure S5:

High-performance liquid chromatograms of (A.) cyanidin, (B.) delphinidin and sacred lotus extracts including (C.) seed embryo, (D.) flower stalk, (E.) stamen, (F.) old leaf, (G.) petal, and (H.) leaf stalk. Retention times (R_t) of phenolics in sacred lotus extracts are indicated at a wavelength of 530 nm.

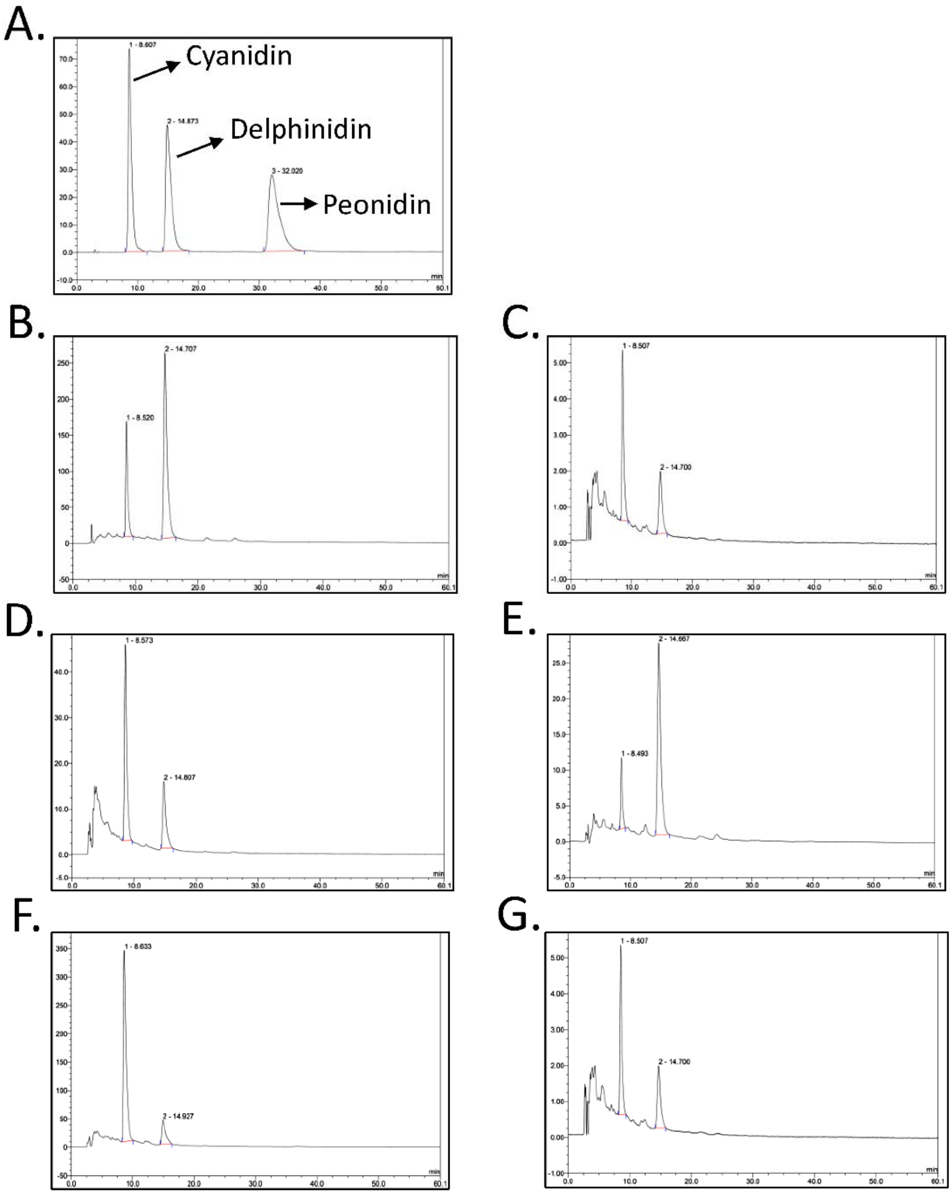

Supplementary Figure S6:

Scheme showed the IC_{50} plots against acetylcholinesterase (AChE) of sacred lotus extracts including (A.) seed embryo, (B.) flower stalk, (C.) stamen, (D.) old leaf, (E.) petal, and (F.) leaf stalk.

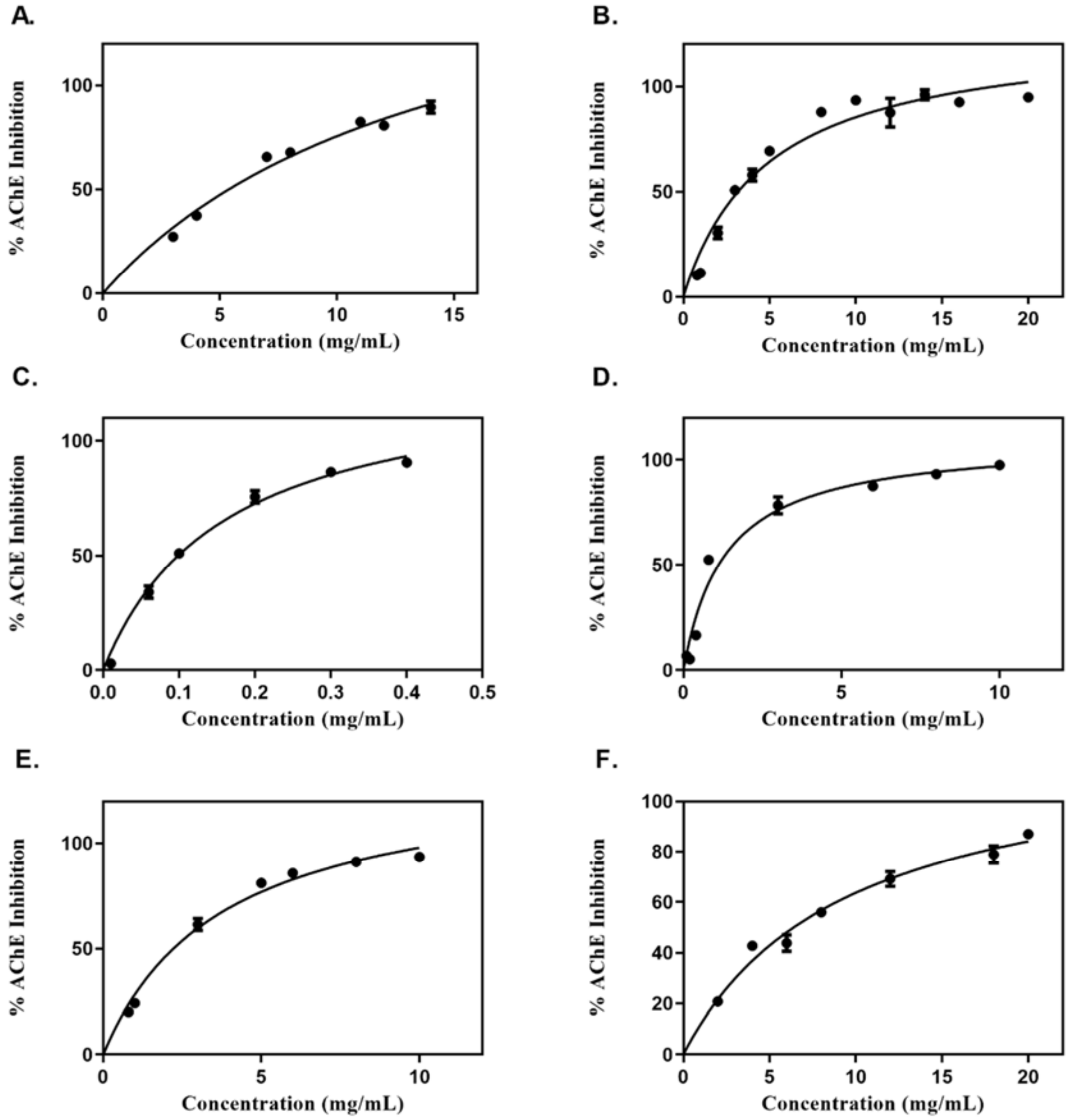

Supplementary Figure S7:

Scheme showing the IC_{50} plots against butyrylcholinesterase (BChE) of sacred lotus extracts including (A.) seed embryo, (B.) flower stalk, (C.) stamen, (D.) old leaf, (E.) petal, and (F.) leaf stalk

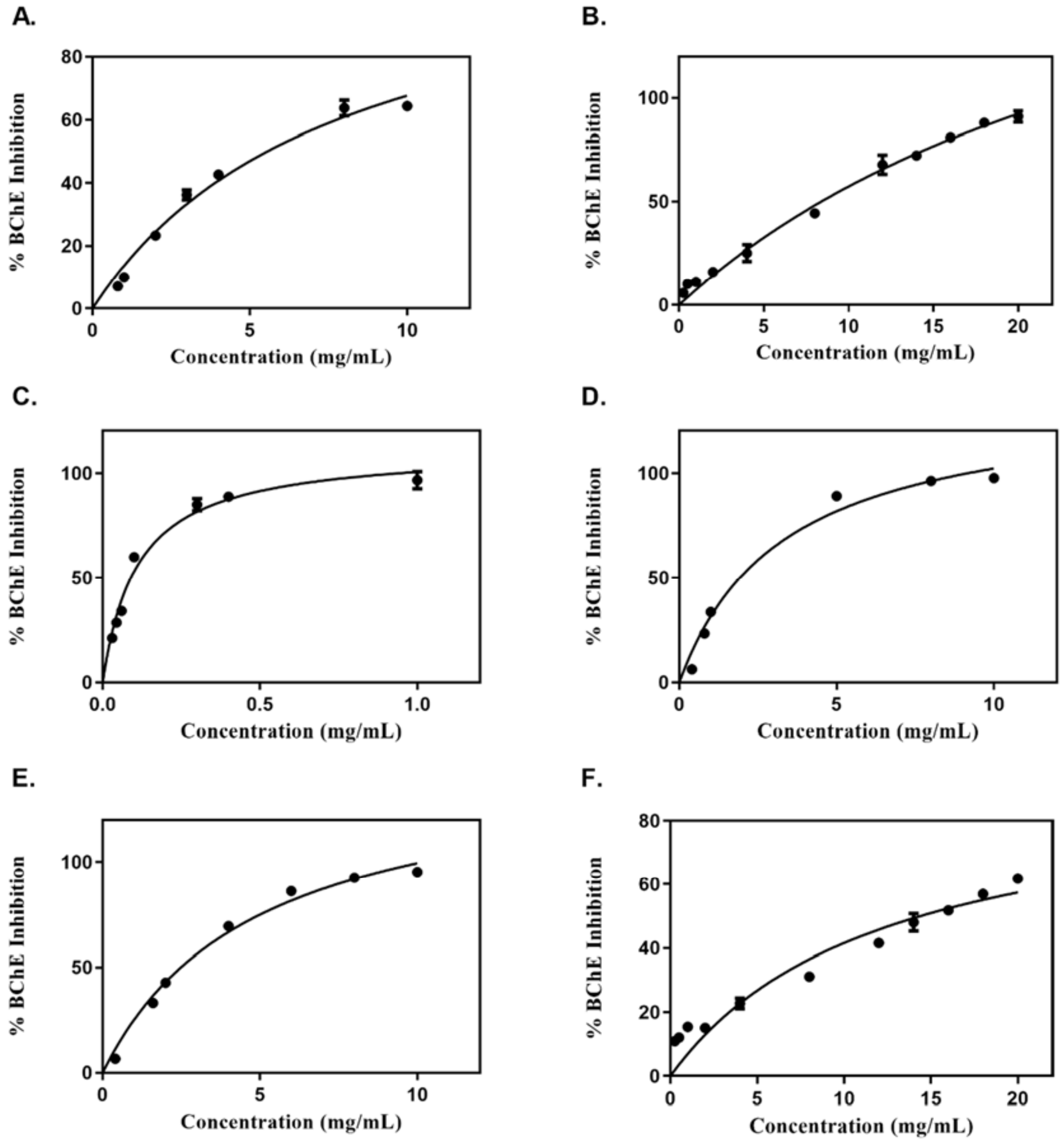

Supplement: Supplementary file 1 [file molecules-25-03713-s001.pdf]
